# Supplementary material for: Molecular programs of fibrotic change in aging human lung
Source: Nat Commun. 2021 Nov 2;12:6309. doi: 10.1038/s41467-021-26603-2 (PMC8563941; doi:10.1038/s41467-021-26603-2)
Supplement: Supplementary file 1 — Supplementary Information [file 41467_2021_26603_MOESM1_ESM.pdf]

Supplementary Information

Molecular programs of fibrotic change in aging human lung

Seoyeon Lee[1]*, Mohammad Naimul Islam[2]*, Kaveh Boostanpour[1], Dvir Aran[3], Guangchun Jin[2], Stephanie Christenson[1], Michael A. Matthay[1], Walter L. Eckalbar[1], Daryle J. DePianto[4], Joseph R. Arron[4], Liam Magee[1], Sunita Bhattacharya[2,5], Rei Matsumoto[6], Masaru Kubota[6], Donna L. Farber[6,7], Jahar Bhattacharya[2]*, Paul J. Wolters[1]*, Mallar Bhattacharya[1]*

[1] Department of Medicine, Division of Pulmonary, Critical Care, Allergy, and Sleep, University of California, San Francisco, CA, USA.

[2] Lung Biology Laboratory, Department of Medicine, Division of Pulmonary, Allergy, and Critical Care Medicine, Vagelos College of Physicians and Surgeons of Columbia University, New York, NY, USA.

[3] Lorry I. Lokey Interdisciplinary Center for Life Sciences & Engineering, Technion Israel Institute of Technology, Haifa, Israel.

[4] Genentech Research and Early Development, Genentech, Inc. South San Francisco, CA, USA.

[5] Department of Pediatrics, Vagelos College of Physicians and Surgeons of Columbia University, New York, NY, USA

[6] Department of Surgery, Vagelos College of Physicians and Surgeons of Columbia University, New York, NY, USA

[7] Department of Microbiology and Immunology, Columbia University, New York, NY, USA

*These authors contributed equally: Seoyeon Lee, Mohammad Naimul Islam; Jahar Bhattacharya, Paul J. Wolters, and Mallar Bhattacharya.

Correspondence and requests for material should be directed to M.B. (mallar.bhattacharya@ucsf.edu), P.J.W. (paul.wolters@ucsf.edu), or J.B. (jb39@columbia.edu)

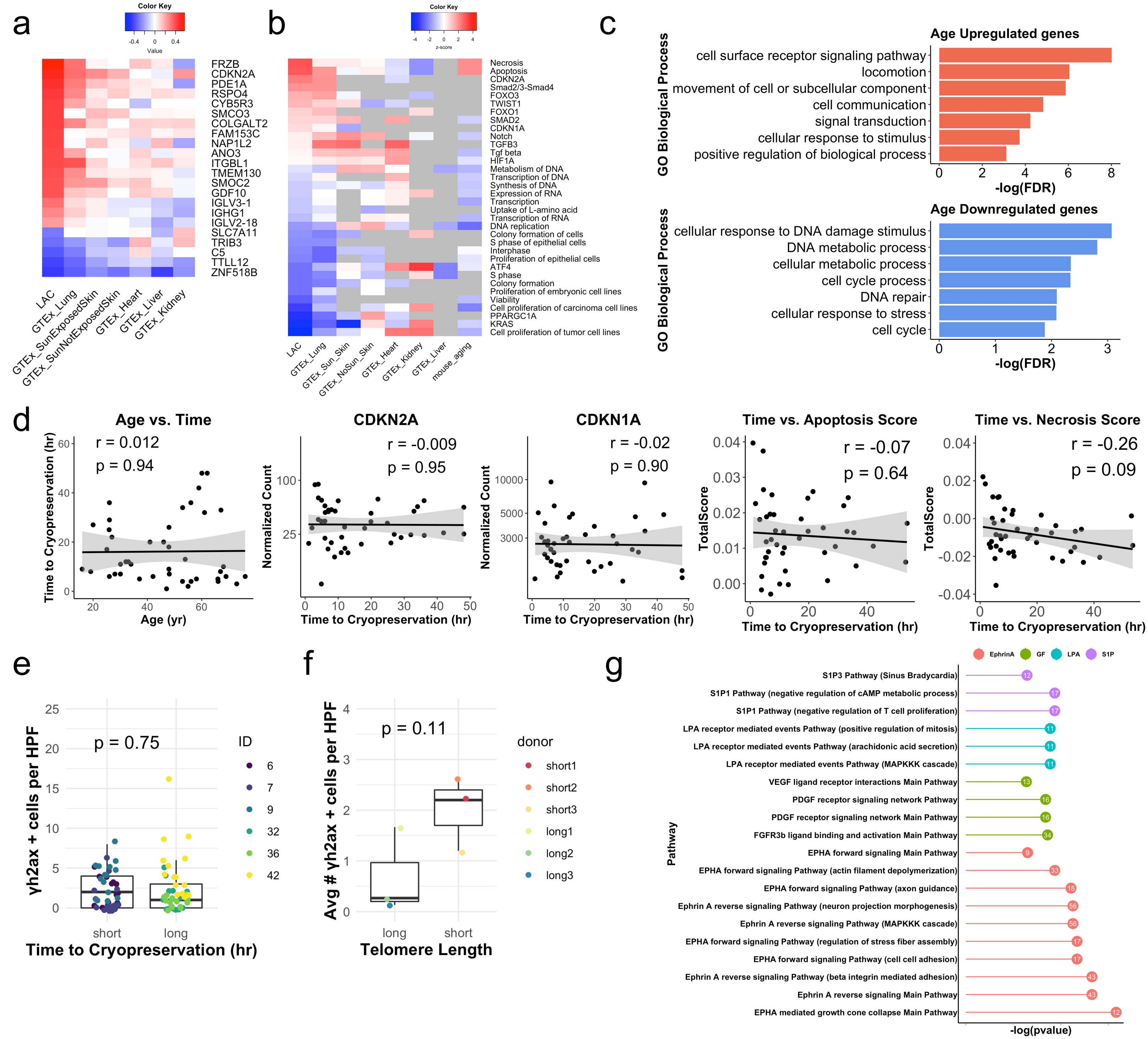

Supplementary Figure 1 **a**, Heatmap of Pearson R between gene expression and age for the LAC and multiple GTEx tissue datasets. **b**, Genes used for IPA in Figure 2c were used to compute IPA results for the same pathways in multiple other datasets. Z scores are shown for pathways reaching significance at p ≤ 0.05. Pathways in grey were not detected or not significant. **c**, Selected pathways from Gene Ontology analysis. Shown pathways were significant (FDR ≤ 0.05) in both LAC and GTEx. –log(FDR) for LAC is shown. **d**, Time between cross-clamp and cryopreservation was plotted against age for LAC samples with available data (N=43 biologically independent samples). Normalized counts for the senescence markers CDKN1A and CDKN2A were plotted against time between cross-clamp and cryopreservation (log scale). For LAC samples with available time data, single sample gene set enrichment scores were calculated for apoptosis and necrosis genes from IPA and plotted against time. Pearson R and P value are shown. The gray bands represent 95% confidence intervals. **e**, γH2AX immunohistochemistry of samples with the shortest and longest times to cryopreservation (N=3 biologically independent samples in each group, N=15 images examined per sample). Each dot represents the number of γH2AX+ cells per image. P value is for a two-sided student's t-test comparing all dots from the short time group versus the long. **f**, Average number of γH2AX+ cells per image computed for each individual analyzed for Figure 2d (N=3 biologically independent samples in each group). P value is for a two-sided Welch's t-test. For boxplots in **e-f**, the middle line shows the median, the lower and upper hinges are the first and third quartiles, and the lower and upper whiskers extend to the value at most 1.5IQR below the first quartile or 1.5IQR above the third quartile, respectively. Data beyond the end of the whiskers are outlying points. **g**, Dot chart of Pathway Activation Levels[31] for profibrotic growth factor pathways that were significantly different between the oldest and youngest quintiles in both LAC and GTEx (P value ≤ 0.05). -log(P value) for LAC is plotted. The value shown within each dot is the difference between the average score for old and young, computed for LAC.

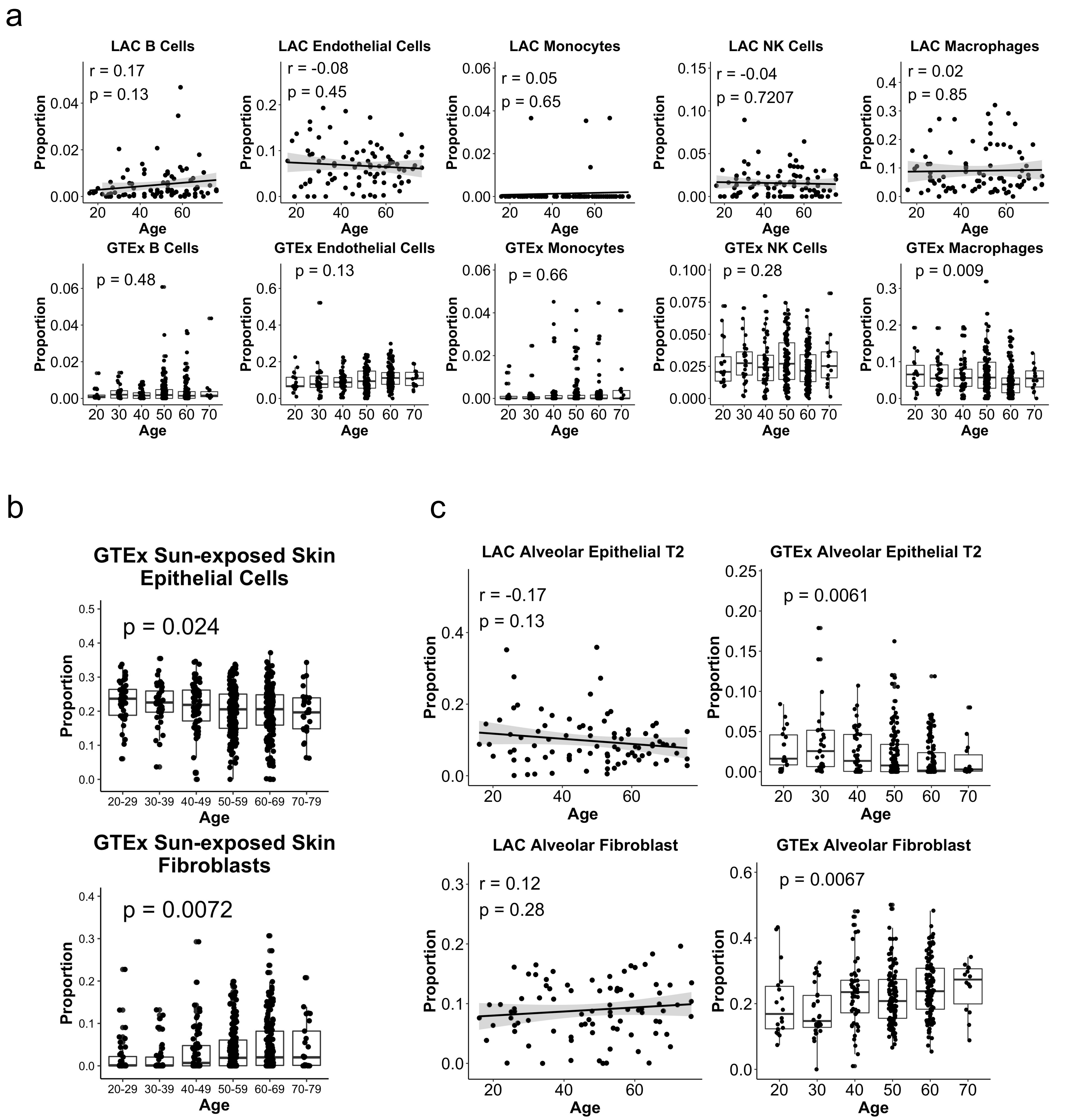

<u>Supplementary Figure 2</u> a, Cell type deconvolution for LAC (N=86 biologically independent samples) and GTEx lung (N=345 biologically independent samples). The gray bands represent 95% confidence intervals. Age for GTEx is represented in decades. **b**, Cell type deconvolution of bulk RNA-seq data from the GTEx sun-exposed skin dataset (N=522 biologically independent samples). **c**, Cell type deconvolution of bulk RNA-seq data from the LAC (N=86 biologically independent samples) and GTEx lung (N=345 biologically independent samples) with cell subtype annotations from Travaglini et al[41]. Pearson R and and P value are shown for LAC, and 1-way ANOVA is shown for GTEx. The gray bands represent 95% confidence intervals. For boxplots in **a-c**, the middle line shows the median, the lower and upper hinges are the first and third quartiles, and the lower and upper whiskers extend to the value at most 1.5IQR below the first quartile or 1.5IQR above the third quartile, respectively. Data beyond the end of the whiskers are outlying points.

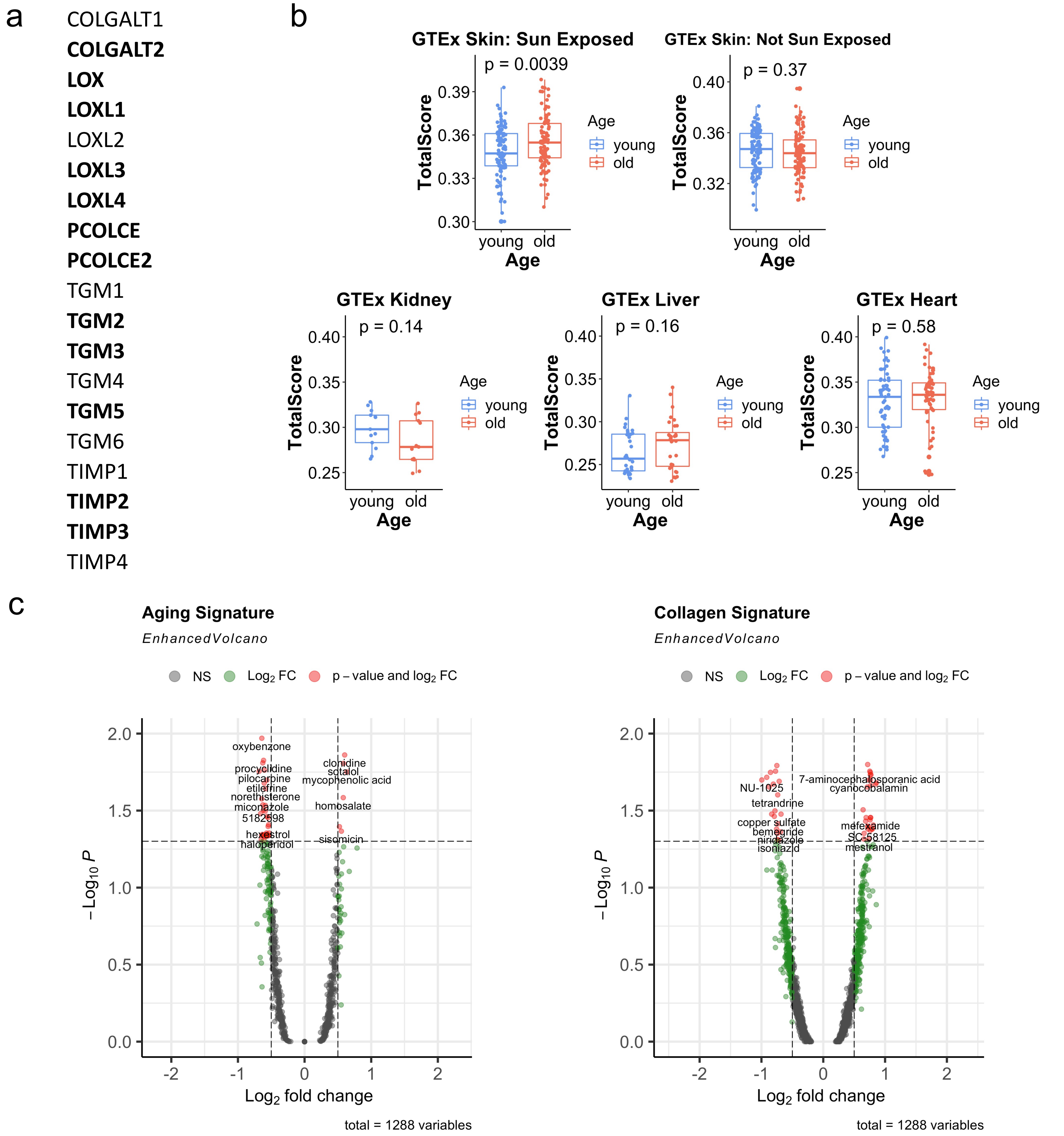

Supplementary Figure 3 **a**, List of collagen processing and cross-linking genes used for the single sample gene set enrichment scoring in Figure 3c (bolded genes were enriched with age in the LAC). **b**, Single sample gene set enrichment analysis for the collagen processing genes enriched in aging from the oldest and youngest quintiles from GTEx sun-exposed skin, non-sun-exposed skin, kidney, liver, and heart (N=104, N=104, N=13, N=32, N=61 biologically independent individuals in each age group, respectively). P values are for two-tailed Student's t-test. For the boxplots, the middle line shows the median, the lower and upper hinges are the first and third quartiles, and the lower and upper whiskers extend to the value at most 1.5IQR below the first quartile or 1.5IQR above the third quartile, respectively. Data beyond the end of the whiskers are outlying points. **c**, Volcano plots of the Connectivity Map analysis indicating compounds predicted to increase or decrease expression of genes from the Lung Aging Signature (Figure 1d) and the Collagen Regulatory Gene Signature (bolded genes in Figure S3a).

Supplementary table 1: Lung donor demographic data in aggregate

UCSF (Figures 1, 2 ,3)

Age		0-29 (n=15)	30-39 (n=13)	40-49 (n=13)	50-59 (n=22)	60-69 (n=16)	70-79 (n=7)
Mean Age (yr)		23.6	33.7	44.9	54.5	64.6	73.3
Male gender (%)		80	54	54	46	69	29
Ethnicity (n)	White	5	6	10	14	13	5
	Black/African American	1	1	1	0	0	0
	Hispanic/Latino	6	5	1	6	3	1
	Asian	3	1	0	1	0	1
	Other	0	0	1	1	0	0
Ever Smoked (%)		60	54	15	50	56	43

Columbia (Figure 4)

Age		18-50 (n=7)	52-86 (n=7)
Mean Age (yr)		35	63
Male gender (%)		57	29
Ethnicity (n)	White	1	3
	Black/African American	2	4
	Hispanic/Latino	3	0
	Asian	1	0
	Other	0	0
Ever Smoked (%)		60	54

Supplementary table 2: Overrepresented GO Pathways

P-values are for Fisher's Exact test. The Benjamini-Hochberg False Discovery Rates were computed.

GO Pathway	LAC FDR	GTEx FDR	Analysis
positive regulation of response to stimulus (GO:0048584)	7.95E-13	2.66E-04	Upregulated genes with age
negative regulation of response to stimulus (GO:0048585)	7.85E-10	6.59E-03	Upregulated genes with age
regulation of response to stimulus (GO:0048583)	1.19E-09	9.13E-09	Upregulated genes with age
cell surface receptor signaling pathway (GO:0007166)	9.65E-09	1.38E-06	Upregulated genes with age
locomotion (GO:0040011)	8.96E-07	4.50E-02	Upregulated genes with age
response to stimulus (GO:0050896)	1.14E-06	1.65E-02	Upregulated genes with age
movement of cell or subcellular component (GO:0006928)	1.33E-06	1.08E-03	Upregulated genes with age
cell communication (GO:0007154)	1.46E-05	5.33E-09	Upregulated genes with age
signaling (GO:0023052)	1.63E-05	3.22E-09	Upregulated genes with age
signal transduction (GO:0007165)	5.76E-05	1.62E-06	Upregulated genes with age
cellular response to stimulus (GO:0051716)	1.86E-04	3.89E-04	Upregulated genes with age
positive regulation of biological process (GO:0048518)	7.61E-04	1.37E-11	Upregulated genes with age
cellular macromolecule metabolic process (GO:0044260)	4.15E-07	8.37E-19	Downregulated genes with age
nucleic acid metabolic process (GO:0090304)	5.74E-07	2.22E-30	Downregulated genes with age
macromolecule metabolic process (GO:0043170)	4.82E-05	2.63E-23	Downregulated genes with age
nucleobase-containing compound metabolic process (GO:0006139)	4.83E-05	3.73E-43	Downregulated genes with age
heterocycle metabolic process (GO:0046483)	5.16E-05	6.26E-47	Downregulated genes with age
cellular nitrogen compound metabolic process (GO:0034641)	5.46E-05	7.41E-60	Downregulated genes with age
cellular aromatic compound metabolic process (GO:0006725)	1.11E-04	1.56E-44	Downregulated genes with age
nitrogen compound metabolic process (GO:0006807)	1.81E-04	7.03E-46	Downregulated genes with age
cellular response to DNA damage stimulus (GO:0006974)	8.48E-04	3.77E-06	Downregulated genes with age
DNA metabolic process (GO:0006259)	1.54E-03	1.03E-09	Downregulated genes with age
primary metabolic process (GO:0044238)	1.84E-03	2.52E-50	Downregulated genes with age
organic cyclic compound metabolic process (GO:1901360)	1.88E-03	5.19E-47	Downregulated genes with age
cellular macromolecule biosynthetic process (GO:0034645)	3.65E-03	7.29E-24	Downregulated genes with age
cellular metabolic process (GO:0044237)	4.53E-03	1.24E-63	Downregulated genes with age
cell cycle process (GO:0022402)	4.60E-03	5.21E-06	Downregulated genes with age
organic substance metabolic process (GO:0071704)	7.06E-03	5.00E-48	Downregulated genes with age
nucleic acid-templated transcription (GO:0097659)	7.20E-03	5.34E-03	Downregulated genes with age
transcription, DNA-templated (GO:0006351)	7.27E-03	5.27E-03	Downregulated genes with age
macromolecule biosynthetic process (GO:0009059)	7.83E-03	1.11E-22	Downregulated genes with age
DNA repair (GO:0006281)	8.05E-03	9.56E-07	Downregulated genes with age
cellular response to stress (GO:0033554)	8.16E-03	1.44E-07	Downregulated genes with age
mitotic cell cycle (GO:0000278)	9.51E-03	4.93E-07	Downregulated genes with age
RNA biosynthetic process (GO:0032774)	1.10E-02	3.45E-03	Downregulated genes with age
chromosome organization (GO:0051276)	1.15E-02	3.42E-05	Downregulated genes with age
mitotic cell cycle process (GO:1903047)	1.16E-02	5.44E-07	Downregulated genes with age
RNA metabolic process (GO:0016070)	1.31E-02	4.38E-23	Downregulated genes with age
cell cycle (GO:0007049)	1.32E-02	4.85E-07	Downregulated genes with age
gene expression (GO:0010467)	1.32E-02	4.31E-28	Downregulated genes with age
macromolecule modification (GO:0043412)	2.24E-02	5.12E-04	Downregulated genes with age

Supplementary table 3: Primer Sequences and PCR Conditions for Telomere Length qPCR

Primer Sequences

Name	Sequence
36B4 L	CAG CAA GTG GGA AGG TGT AAT CC
36B4 R	CCC ATT CTA TCA TCA ACG GGT ACA A
q Telo L	CGG TTT GTT TGG GTT TGG GTT TGG GTT TGG GTT TGG GTT
q Telo R	GGC TTG CCT TAC CCT TAC CCT TAC CCT TAC CCT TAC CCT

qPCR Conditions

Stage	Temperature	Time
Hold	95°C	15min
PCR (40 cycles)	94°C	15sec
	60°C	1min
Melt Curve	95°C	15sec
	60°C	1min
	95°C	15sec
