# Supplementary material for: Molecular programs of fibrotic change in aging human lung
Source: Nat Commun. 2021 Nov 2;12:6309. doi: 10.1038/s41467-021-26603-2 (PMC8563941; doi:10.1038/s41467-021-26603-2)
Supplement: Supplementary file 3 — Description of Additional Supplementary Files [file 41467_2021_26603_MOESM3_ESM.docx]

**File Name: Supplementary Data 1**

**Description**: Results from Ingenuity Pathway Analysis (IPA) computed with the genes from LAC that were correlated with age at p ≤ 0.05 level of significance. Upstream regulators and disease and functions with p ≤ 0.05 are shown. P values are for right-tailed Fisher’s exact test without adjustment for multiple comparisons.

**File Name: Supplementary Data 2**

**Description**: Results from Pathway Activation Level Analysis comparing the oldest and youngest quintiles in the LAC and GTEx lung. Pathways with p ≤ 0.05 are shown. P values are for two-sided student’s t-test without adjustment for multiple comparisons. For each pathway, the difference between the average score for old and young was computed for both LAC and GTEx.

**File Name: Supplementary Data 3**

**Description**: Markers for human lung epithelial cells from GSE122960 determined by differential expression analysis. P values are for non-parametric Wilcoxon rank sum tests. Adjusted p values are based on Bonferroni correction.

**File Name: Supplementary Data 4**

**Description**: Markers for human lung fibroblasts from GSE122960 determined by differential expression analysis. P values are for non-parametric Wilcoxon rank sum tests. Adjusted p values are based on Bonferroni correction.

**File Name: Supplementary Data 5**

**Description**: Results from drug perturbation analysis using the Connectivity Map dataset. Connectivity scores were computed for the 22 aging genes (Figure 1d) and the 12 collagen processing and cross-linking genes enriched with age in the Lung Aging Cohort (Supplementary Figure 3a, bolded genes). Significance of the connectivity was determined by permutation testing.
