# Supplementary material for: Molecular programs of fibrotic change in aging human lung
Source: Nat Commun. 2021 Nov 2;12:6309. doi: 10.1038/s41467-021-26603-2 (PMC8563941; doi:10.1038/s41467-021-26603-2)
Supplement: Supplementary file 9 — Reporting Summary [file 41467_2021_26603_MOESM9_ESM.pdf]

Corresponding author(s): Mallar Bhattacharya, Paul Wolters, Jahar Bhattacharya

Last updated by author(s): Sep 21, 2021

Reporting Summary

Nature Research wishes to improve the reproducibility of the work that we publish. This form provides structure for consistency and transparency in reporting. For further information on Nature Research policies, see our Editorial Policies and the Editorial Policy Checklist.

Statistics

For all statistical analyses, confirm that the following items are present in the figure legend, table legend, main text, or Methods section.

n/a	Confirmed	
☐	☒	The exact sample size (*n*) for each experimental group/condition, given as a discrete number and unit of measurement
☐	☒	A statement on whether measurements were taken from distinct samples or whether the same sample was measured repeatedly
☐	☒	The statistical test(s) used AND whether they are one- or two-sided<br>*Only common tests should be described solely by name; describe more complex techniques in the Methods section.*
☐	☒	A description of all covariates tested
☐	☒	A description of any assumptions or corrections, such as tests of normality and adjustment for multiple comparisons
☐	☒	A full description of the statistical parameters including central tendency (e.g. means) or other basic estimates (e.g. regression coefficient) AND variation (e.g. standard deviation) or associated estimates of uncertainty (e.g. confidence intervals)
☐	☒	For null hypothesis testing, the test statistic (e.g. *F*, *t*, *r*) with confidence intervals, effect sizes, degrees of freedom and *P* value noted<br>*Give P values as exact values whenever suitable.*
☒	☐	For Bayesian analysis, information on the choice of priors and Markov chain Monte Carlo settings
☒	☐	For hierarchical and complex designs, identification of the appropriate level for tests and full reporting of outcomes
☐	☒	Estimates of effect sizes (e.g. Cohen's *d*, Pearson's *r*), indicating how they were calculated

Our web collection on statistics for biologists contains articles on many of the points above.

Software and code

Policy information about availability of computer code

Data collection	No software was used.
Data analysis	The following R and Python packages and software was used: DESeq2 (1.30.1), Seurat (4.0.1), SingleR (1.4.1), MuSiC (0.1.1), Singscore (1.10.0), PharmacoGx (2.2.4), DOSE (3.16.0), GSEA (Broad, 4.1.0), PANTHER (version 14), Oncoboxlib (1.2.3), CMAP (Lamb et al., 2006), and IPA (Qiagen, IPA Spring Release (March 2020)). Codes used for implementation of the packages, statistical tests, and figure generation are available on: <https://github.com/jasminelee63/AgingLungBulkRNASeq>.

For manuscripts utilizing custom algorithms or software that are central to the research but not yet described in published literature, software must be made available to editors and reviewers. We strongly encourage code deposition in a community repository (e.g. GitHub). See the Nature Research guidelines for submitting code & software for further information.

Data

Policy information about availability of data

All manuscripts must include a data availability statement. This statement should provide the following information, where applicable:

- Accession codes, unique identifiers, or web links for publicly available datasets
- A list of figures that have associated raw data
- A description of any restrictions on data availability

Processed RNA-seq data have been deposited in de-identified fashion at NCBI GSE165192 <https://www.ncbi.nlm.nih.gov/geo/query/acc.cgi?acc=GSE165192> and are publicly available. FASTQ files will be accessible on request from the NCBI Sequence Read Archive dbGAP under phs002484.v1.p1 <http://www.ncbi.nlm.nih.gov/projects/gap/cgi-bin/study.cgi?study_id=phs002484.v1.p1>. Human lung single cell data used for cell type deconvolution analysis were downloaded from GSE122960 <https://www.ncbi.nlm.nih.gov/geo/query/acc.cgi?acc=GSE122960> and Synapse <https://www.synapse.org/#!

Synapse:syn21041850>. Skin single cell data was downloaded from GSE147424 <https://www.ncbi.nlm.nih.gov/geo/query/acc.cgi?acc=GSE147424>.

Field-specific reporting

Please select the one below that is the best fit for your research. If you are not sure, read the appropriate sections before making your selection.

[x] Life sciences [ ] Behavioural & social sciences [ ] Ecological, evolutionary & environmental sciences

For a reference copy of the document with all sections, see nature.com/documents/nr-reporting-summary-flat.pdf

Life sciences study design

All studies must disclose on these points even when the disclosure is negative.

Sample size	We determined sample size based on expected effect size and variability within the sample.
Data exclusions	Samples were not excluded in the study with the exception of qPCR quantitation of telomere length, where, prospectively, samples with standard deviation of triplicates higher than 0.25 were excluded (2 of 86 samples were excluded based on these quality-control criteria).
Replication	All experiments with the exception of RNA-seq were performed with at least 3 technical replicates. While we were not able to perform technical replicates for RNA-Seq, instead we validated our results in a separate cohort (GTEx).
Randomization	Randomization was not applicable because our study applied bulk RNA-seq to all samples.
Blinding	Investigators were blinded to sample identity during data collection and analysis.

Reporting for specific materials, systems and methods

We require information from authors about some types of materials, experimental systems and methods used in many studies. Here, indicate whether each material, system or method listed is relevant to your study. If you are not sure if a list item applies to your research, read the appropriate section before selecting a response.

Materials & experimental systems

n/a	Involved in the study	
[ ]	[x]	Antibodies
[ ]	[x]	Eukaryotic cell lines
[x]	[ ]	Palaeontology and archaeology
[x]	[ ]	Animals and other organisms
[ ]	[x]	Human research participants
[x]	[ ]	Clinical data
[x]	[ ]	Dual use research of concern

Methods

n/a	Involved in the study	
[x]	[ ]	ChIP-seq
[x]	[ ]	Flow cytometry
[x]	[ ]	MRI-based neuroimaging

Antibodies

Antibodies used	Purified anti-H2AX-Phosphorylated (Ser139) Antibody, Biolegend, 613402, Clone 2F3, B158027. Anti-proSPC Antibody, EMD Millipore, AB3786. Alexa Fluor 594-conjugated Secondary Antibody, Life Technologies, A21207.
Validation	Biolegend 613402: validated antibody known to react with human species. Manufacturer's website lists published examples of IHC on human tissue.<br>EMD Millipore AB3786: validated antibody known to react with human species. Manufacturer's website lists published examples of IF on human tissue. We have also validated both antibodies by Western Blot in previous studies.

Eukaryotic cell lines

Policy information about cell lines

Cell line source(s)	Gift of Elizabeth Blackburn. Three cell lines were used: T47D, DU4475, HCC1806. (PMID: 23610451)
Authentication	The cell lines were used as controls for telomere length analysis, and their telomere lengths have been independently validated by Flow-FISH.
Mycoplasma contamination	Cell lines were not tested for Mycoplasma contamination.

Commonly misidentified lines (See ICLAC register)	N/A

Human research participants

Policy information about studies involving human research participants

Population characteristics	Deceased donor lungs were used for this study. Population characteristics are listed in our Supplementary table 1, and includes age, gender, ethnicity, and smoking status.
Recruitment	Lungs were acquired from the organ donor networks, Donor Network West and LiveOnNY.
Ethics oversight	Lungs were obtained from brain-dead (deceased) individuals, and thus this study does not qualify as human subjects research, as confirmed by the UCSF and Columbia University IRBs and according to United States Department of Health and Human Services human subject regulations under 45 CFR 46.

Note that full information on the approval of the study protocol must also be provided in the manuscript.
